# Bibliography

**Published:** 1903-12

**Authors:** 


### Bibliography.

Principles and Practice of crowning Teeth. With four hundred and fifty-nine illustrations. By Hart J. Goslee, D.D.S., Chicago, Ill., Professor of Prosthetic Dentistry and Crown- and Bridge-Work, Chicago College of Dental Surgery, etc. The Consolidated Dental Manufacturing Company, New York; Claudius Ash & Sons (Limited), London, 1903.

The author says, "In assuming to supply such a possible need (a modern text- and reference-book) an effort has been made to present the subject matter in a practical and concise form and in a more or less systematic and sequential order; as well as to avoid, in so far as possible, any consideration of methods which may have proved, or which are deemed to be, impracticable."

That the author has succeeded in this is made apparent to any one carefully examining this book. There has been so much written in periodicals in regard to bridge-work and crowns that the inexperienced operator feels a natural timidity in undertaking the work, and a large proportion of practitioners, when they do attempt it, make but a poor showing, either through lack of skill or a minimum of knowledge. There has been nothing ever introduced into dentistry that has been so much abused as this procedure. This has, possibly, been due to the lack of practical knowledge of metal work, that went out with the advent of rubber, coupled with knowledge of pathological principles of what must be done or left undone in this work. It is, therefore, with peculiar satisfaction that this book has been given to the profession of dentistry as a text-book and reference work. The author's name is a guarantee for good work, and this is borne out by the care with which every process, proved to have value, is demonstrated.

It is not always that illustrations are satisfactory, but the large number given fully show in every detail the descriptive text. It would, therefore, seem that one wholly unacquainted with the process could, by following the author, work up a practical ability sufficient for all cases coming under his care.

The author, it is thought, has made a mistake in discarding any lengthy allusion to therapeutic treatment. It is just here wherein the greatest danger lies and while it is supposed that all graduates of dental colleges are properly taught how to treat teeth and how to handle bands in connection with the pericementum, it is very evident, from the fearful examples coming under observation, that proper precautions are either neglected, through ignorance or something much more to be condemned.

The author's statement on devitalization of pulps, "It now must generally be conceded to be a safer precaution, in a great majority of cases, to destroy such pulps as a prophylactic procedure, as well as to facilitate the necessary mechanical preparation when the crown is to entirely cover the end of the root," seems to the writer to be bad advice. While it is true that violent irritations may follow the preparation of teeth for crowning through thermal changes, causing shock to the pulp, the author should recognize that the pulp is something more than "a purely formative organ," and that its "physiological function" does not terminate with the complete development of the tooth. Teeth may last for an uncertain period without pulps, but no tooth is equally resistant to destructive influences without it as with it in full vitality.

It is somewhat surprising that no allusion is made to the tooth so universally used, at one period, for the insertion of a wooden pivot. The author seems to think that the English tube teeth were exclusively used for this purpose. While these were probably used to some extent with wooden pivots, the real wooden pivot tooth was manufactured extensively in this country without the metal tube. For ordinary pivoting the wooden pivot was generally used.

Another similar error, on page 8, is in giving credit to Dr. M. H. Webb "for swaging or burnishing a metal plate to the end of the root, then perforating it to admit of inserting into the canal a dowel which was soldered to the plate." Is it not about time that writers on dental subjects should cease to imagine that modern dentistry began, if it did not end, with Webb? It would require more years than the writer has at command to go back in memory to the time when this method was not in existence, and Webb simply adopted an old plan of inserting teeth.[1]

When properly prepared and inserted, a bridge is of great value, but improperly made and inserted without judgment, it is

---

[1] See Harris, Principles and Practice, fourth edition, p. 645, 1850.

one of the worst artificial substitutes that dentistry has ever produced.

The publishers are to be congratulated on the excellence of the work, as to its general character, but more especially in the beauty and effectiveness of the illustrations.

Archinard's Bacteriology. A Manual for Students and Physicians. By P. E. Archinard, M.D., of Tulane University Medical Department, New Orleans. In one 12mo volume of two hundred and ten pages, with seventy-four illustrations. Lea Brothers & Co., Publishers, Philadelphia and New York, 1903.

This book is one of a series which the publishers decided "should embrace the entire realm of medicine; that the individual volumes should authoritatively cover their subjects in all essentials."

This one of the series, as its title indicates, is upon the subject of "Microscopy and Bacteriology." The introductory chapter briefly, but very clearly, treats of the "Refraction of Light and the Microscope," its use and care. The first chapter deals, very properly, with the history of bacteriology and its fundamental principles, followed by Chapter II., on the examination of bacteria, staining, etc. These are followed by chapters on the processes, media, and utensils for the cultivation of bacteria; inoculation of culture-media; sterilization, inoculation of animals, infection and immunity, pathogenic bacteria, etc. This will give a general idea of the scope of this small book, but its real value consists in the able condensation of the matter to be found in larger works devoted to this important subject. The book is well illustrated with engravings.

The idea that usually accompanies the examination of a compend is that they are unsatisfactory and lead to superficial knowledge. While this is generally true, it does not apply to this book or to any of the Pedersen series, for they constitute really a series of text-books that students can readily carry in their pockets as convenient refreshers of memory.

Each part is followed by a series of questions. It may be doubted whether these are of much value, either to the quiz-master or to the student for self-examination.

Modern Materia Medica and Therapeutics. By A. A. Stevens, A.M., M.D., Lecturer on Physical Diagnosis in the University of Pennsylvania, etc. Third edition, entirely rewritten. W. B. Saunders & Co., Philadelphia, New York, London, 1903.

The author of this work says, in his preface to this the third edition, "Instead of considering the drugs in alphabetical order, as in previous editions, he has thought it best in the present revision to classify them according to their pharmacologic action." This plan may be of advantage in some directions, but it leads necessarily to cross reference, and this is of special annoyance in the study of a drug. The tendency to prepare works upon materia medica on this plan, instead of making the study more satisfactory to the student, has a tendency to a reverse effect. To the practitioner, however, the present method presents many advantages, and hence will be regarded by that class as a decided improvement over the earlier editions.

Aside from this seeming defect, there can be no criticism upon the general treatment of drugs in their physiologic action and therapeutics. The author has not only included nearly all the recently introduced drugs, but has clearly designated their action upon the system, thus giving the general practitioner a very certain guide for their proper use. A minor criticism may be made as to the very brief statement of the origin of drugs and the processes adopted to produce these. This, however, is a fault common to most works on materia medica outside of the large authorities, which necessarily enter minutely into this matter. It is doubtless supposed that practitioners are familiar with origin and processes, and students will naturally refer to larger works for more detailed descriptions.

In treating of arsenic, under the head of Escharotics, the author says of it, "Its *modus operandi* is not definitely known, but, as it does not combine chemically with the tissues, it is supposed to act directly upon the cells in a specifically poisonous manner." The experiments of Miller, Berlin, have demonstrated that the arsenic acts to paralyze the sensory nerves, and in this way it cuts off nutrition and ends eventually in the destruction of the tissue. It, however, is not a true escharotic in the sense of cauterizing the tissue, as zinc chloride and other true escharotics would do.

The experience of dentists clinically, and few have larger opportunities of observing the action of this drug, has been to confirm Miller's deductions. They have, by long and constant observation, concluded that in the treatment of pulps the action of arsenic is first to paralyze, and then devitalization slowly follows.

In describing the physiologic action of silver-nitrate, the author says of it: "Its corrosive action, however, never extends very deeply on account of the impenetrable nature of the coagulum that is at once formed." This is a mistake that should be corrected. It is the old story, often repeated, and which has been proved over and over again to be unture. Perhaps dentistry can give the key to the action of this escharotic, for in the treatment of teeth with this agent it has been observed that it penetrates deeply the tubuli of the dentine, the depth of penetration depending solely upon the strength and quantity used, the coagulum having no deterrent quality. The reviewer several years since settled the question, through laboratory experiments, that silver nitrate was one of the most deeply penetrating coagulants and that the coagulum furnished no bar to its progress.

The part of the book devoted to "Remedial Measures other than Drugs" covers the use of electricity, massage, movement therapy, Nauheim treatment, cold, heat, etc. The closing portion of the book, covering one hundred and thirty-five pages, is devoted to "Applied Therapeutics," and will be a very valuable portion to both practitioners and students in medicine, but to the dentist it has a special value in contributing to that general knowledge so important for all workers in any specialty of the healing art.

Taking the book as a whole it must be regarded as an improvement on the author's previous work, and in many respects superior to the average books treating upon the same subject.

It is prepared in the usual careful manner of the Saunders publication company.

